# Reply: Lack of evidence supporting a role for *DPP6* sequence variants in Alzheimer’s disease in the European American population

**DOI:** 10.1007/s00401-021-02277-4

**Published:** 2021-02-16

**Authors:** Rita Cacace, Christine Van Broeckhoven

**Affiliations:** 1 VIB Center for Molecular Neurology, Antwerp, Belgium; 2 Department of Biomedical Sciences, University of Antwerp, Antwerp, Belgium

We appreciate the work performed by Kirola et al. [3] in attempting to replicate our genetic findings in *DPP6* [1]. Kirola et al. investigated association of *DPP6* with Alzheimer’s disease (AD) in whole exome sequencing (WES) data of three cohorts of non-Hispanic white patients and matched controls [3]. Two cohorts included late-onset AD patients: FASe, with 1212 patients and 341 controls and ADSP with 5656 patients and 4601 controls. The third cohort included 1385 early-onset (EO)AD patients and 3864 controls. The authors conclude that there is no association of *DPP6* with AD, not with single variants or in gene burden tests. [3]. The authors hypothesize a population specific effect of *DPP6* in our study [3]. We read the work of Kirola et al. with great interest, but it seems to us that their interpretation of the data is not providing a comprehensive vision of the genetic role of *DPP6* in AD.

We discovered *DPP6* in an AD family linked to chromosome 7q36 [1, 6]. Using whole genome sequencing (WGS), we identified a chromosomal inversion disrupting the *DPP6* coding sequence, leading to haploinsufficiency as disease mechanism [1]. Kirola et al. used WES data which is a limiting factor for observing structural variants [3]. We also screened *DPP6* in AD patients with an onset age ≤ 70 years and in patients with frontotemporal dementia (FTD).We identified an enrichment of rare non-synonymous variants (nonsense, frameshift and missense) in the EOAD cohort (*p* value = 0.03, OR = 2.21 95% CI 1.05–4.82), and the FTD cohort (*p *value = 0.006, OR = 2.59, 95% CI 1.28–5.49) [1]. We applied a SKAT-O rare variant gene-based test, accounting for pathogenic, non-pathogenic, and even protective variants in our statistical model as it is widely accepted that also causal genes harbor benign variants [2]. It is not unlikely that some of the variants identified by Kirola et al. are benign. We uncovered the benign effect of the p.Ala778Thr, which we [1] and Kirola et al. [3] found in patients and controls. Regrettably, the overlap of rare non-synonymous variants between the two studies is limited, seven in the ADSP and none in the EOAD cohorts as we identified a p.Ala655Val [1] instead of the p.Ala655Thr that Kirola et al. reported [3]. Also, the lack of overlap of variants in exon 1, might be consistent with a lower quality of the WES reads in this region. We used Sanger sequencing to overcome this issue [1]. Further, it is not clear how the single variants were selected for inclusion or exclusion from the burden tests (Tables S2 and S3 versus Table S1 in the paper of Kirola et al. [3]). It seems to us that the inclusion criteria were not entirely followed.

Based on our results in the *DPP6* family [1], we predict that loss of function (LoF) variants (structural variants e.g., inversion; mutations leading to premature termination codons (PTCs)), present with a higher penetrance and disease impact, contributing to DPP6 haploinsufficiency. But LoF mutations are extremely rare compared to missense mutations, which might have a variable risk contribution to disease depending on DPP6 expression levels [1]. Kirola et al. identified in *DPP6* a frameshift variant, p.His431fs, and a variant in a donor splice-site (hg19 g.154664404G > A), affecting all protein coding DPP6 isoforms [3]. The variants were found in two AD patients in the ADSP cohort and never in 8806 controls pooled from the three control cohorts. These findings of Kirola et al., support the predicted functional mode of action of *DPP6* [1]. Notably, the p.His431fs appears to be excluded from both burden tests (MAF ≤ 1% and CADD ≥ 20) and is absent from Tables S2-S3 even though it has a MAF ≤ 1% and we calculated a CADD score of 32 for it. Also, the donor splice-site variant is included only in the burden test of variants with a CADD score ≥ 20 (Table S3) while the variant has a MAF ≤ 1% [3].

To complete, the genetic association of *DPP6* with FTD is shown in an independent study which analyzed WGS data of individuals from 23 different sites from Europe, North America, and Australia [5], arguing against a population effect in Belgium. Also, in the latter study, a truncating mutation in DPP6 was observed in one patient, leading to partial loss of DPP6 expression in the brain [5]. We agree that replication of the rare variant association in independent cohorts is fundamental, but we stress that functional evidence is crucial in case of novel disease genes, to understand how they contribute to a specific phenotype. A recent work suggests a neuroprotective role for DPP6 [4]. In aged Dpp6 knockout mice the loss of protein is shown to lead to the enhanced formation of abnormal, enlarged presynaptic terminals [4]. These structures partly colocalize with proteins associated with AD, including Aβ, pTau and chromogranin A [4].

We conclude that the presented work by Kirola et al. shows a lack of replication of our study, but cannot conclude that there is no association of *DPP6* with AD [3], since their study is limited to statistical analysis of rare coding variants without further interpretation of a potential functional effect and the type of variants.
